# Utility of COVID-19 Seropositive Plasma as Convalescent Plasma: An Immune and Neutralization Antibody Seroprevalence Analysis in Blood Donors for Future Potential Pandemic Readiness

**DOI:** 10.7759/cureus.57149

**Published:** 2024-03-28

**Authors:** Ashish Jain, Gita Negi, Daljit Kaur, Vivekanandhan S, Vartika Saxena

**Affiliations:** 1 Transfusion Medicine, All India Institute of Medical Sciences, Rishikesh, Rishikesh, IND; 2 Biochemistry, All India Institute of Medical Sciences, Rishikesh, Rishikesh, IND; 3 Community and Family Medicine, All India Institute of Medical Sciences, Rishikesh, Rishikesh, IND

**Keywords:** pandemic, neutralization antibodies, seroprevalence, immune response, convalescent plasma, covid-19

## Abstract

Objectives: To analyze the seroprevalence of SARS-CoV-2 IgG antibodies and neutralizing antibodies in blood donors during the second wave of the pandemic and to explore the utility of COVID-19 seropositive plasma as convalescent plasma.

Materials and methods: In this study, 696 blood donors were tested for anti-SARS-CoV-2 IgG antibodies using a chemiluminescence assay. By blinding, 271 samples were chosen randomly for testing of neutralizing antibodies by enzyme-linked immunosorbent assay (ELISA) in duplicate among the 696 blood donors tested for anti-SARS-CoV-2 IgG antibodies, irrespective of the positivity or negativity of the result of the anti-SARS-CoV-2 IgG antibodies by chemiluminescence assay. IgG antibody levels were analyzed in signal-to-cutoff (S/Co), while neutralizing antibody levels were analyzed in percentage inhibition.

Results: The seroprevalence of IgG antibodies based on the S/Co for the positive results ≥ 1.00 was 82.75%, while the seroprevalence of neutralizing antibodies based on the percentage inhibition for the positive results ≥ 30% was 89.59%. Frontline workers (FLWs) and Covishield-vaccinated individuals showed higher levels of the anti-SARS-CoV-2 IgG antibodies regarding higher S/Co. In comparison, levels of neutralization antibodies regarding percentage inhibition were higher only in FLWs. Covishield-vaccinated donors elicited a statistically higher seroprevalence of anti-SARS-CoV-2 IgG antibodies compared to the Covaxin-vaccinated, while the seroprevalence of neutralizing antibodies was not statistically different among this group. There was a positive correlation (0.762) between anti-SARS-CoV-2 IgG antibodies and neutralizing antibodies, and almost all donors' of S/Co ≥ 9.5 had neutralizing antibodies.

Conclusion: This study showed higher seroprevalence in the blood donor population compared to published seroprevalence in India's second wave of the pandemic. In the current study, 328 donors (47.12%) of the 696 screened donors were neither vaccinated nor had previous SARS-CoV-2 infection, but many had antibodies. The seroprevalence of neutralizing antibodies (96.42%) was higher than the seroprevalence of the anti-SARS-CoV-2 IgG antibodies (85.71%) in the donors who had previous infection of COVID-19. On the other hand, vaccinated donors showed similar immune responses for neutralizing antibodies and the anti-SARS-CoV-2 IgG antibodies. Higher IgG immune reactivity in S/Co showed a good correlation with neutralizing antibodies and can be used to screen whole blood donors for convalescent plasma donations.

## Introduction

Many therapies were investigated for COVID-19 treatment, including artificially acquired passive immunity, namely, (a) convalescent plasma (CP), (b) pooled human immunoglobulin (Ig) for intravenous or intramuscular administration, (c) high-titer human Ig, and (d) polyclonal or monoclonal antibodies. CP initially showed promising results, as seen in published case reports and series; however, studies published later in the pandemic failed to show its effectiveness [1]. No randomized control trials were available to show its effectiveness in the pandemic's initial part. The National Blood Transfusion Council, Ministry of Health and Family Welfare, Government of India released a guidance document in the first wave and declared it an off-label therapy [2]. The apheresis technique advised CP collection from known reverse transcription-polymerase chain reaction (RT-PCR)-positive donors with a recommendation to test antibody levels. Simultaneously, the Indian Council of Medical Research (ICMR) conducted one of the most extensive clinical trials on 464 moderately ill laboratory-confirmed COVID-19 patients (PLACID trial) [3]. It was concluded that CP therapy did not reduce progression to severe COVID-19 or all-cause mortality in the group that received CP therapy compared to the group that did not. It was also speculated that there would be fewer COVID-19 patients when using CP with a low concentration of specific antibodies against SARS-CoV-2 [1]. Recently, the Association for the Advancement of Blood & Biotherapies (AABB) updated its toolkit to provide information on collecting CP from recovered individuals even after vaccination if they fit the criteria [4]. Studies have shown that neutralizing antibodies rather than antibodies against the spike protein are effective in treatment [5,6]. The present study attempts to establish a correlation between the antibodies against the spike protein and neutralizing antibodies.

Practically, CP therapy is dependent on many factors: finding a convalescing plasma donor having neutralization antibodies capable of neutralizing virus in the recipient, an altruistic attitude toward donation, an equipped apheresis and antibody testing facility in the center, fitness for apheresis procedure, overcoming the travel restrictions in the pandemic, and financial affordability of the patient of apheresis CP-collected product, etc. For a proper randomized control trial, above all factors must be overcome so that a trial's general principles of affordability, availability, and feasibility can be met [7]. Thus, a general query arose about why plasma obtained from donated blood for transfusion of other components could not be used as CP if it had sufficient antibody level. Therefore, another focus of this study was to explore the feasibility of using donated whole blood-derived plasma as CP.

## Materials and methods

This was a cross-sectional study on donors coming to the blood center to donate blood. Ethical approval was taken from the institute's ethics committee (approval number AIIMS/IEC/21/353 dated 11/06/2021). This study was done between June 2021 to July 2021. During the study period, consecutive sampling was done, blood donors who gave consent for this study were enrolled, and samples were taken for antibody testing. The serum samples of the donors were tested for anti-SARS-CoV-2 IgG antibodies against the spike protein by semiquantitative enhanced chemiluminescence-based VITROS equipment (Ortho Clinical Diagnostics, Raritan, NJ). This equipment was validated by using known COVID-19 seropositive samples as positive control samples and running stored serum samples for some other purpose of previous years much before the start of the pandemic as negative control samples. Negative and positive control samples were run with each run. The reactive sample's signal-to-cutoff (S/Co) ratio was 1.00. The total number of samples tested was 696. These serum samples were separated per the standard procedure and stored in a deep freezer at -80 °C for further study. By blinding, 271 samples were chosen randomly for testing of the neutralizing antibodies by enzyme-linked immunosorbent assay (ELISA) in duplicate amongst the 696 blood donors tested for anti-SARS-CoV-2 IgG antibodies, irrespective of the positivity or negativity of the result of the anti-SARS-CoV-2 IgG antibodies by chemiluminescence assay. The principle of detection of neutralization antibodies was semiquantitative detection of neutralizing antibodies against SARS-CoV-2 by the Microwell ELISA (J. Mitra & Co. Pvt. Ltd., New Delhi, India) test, which is an enzyme immunoassay based on blocking ELISA. These neutralizing antibodies developed against SARS-CoV-2 in human plasma prevent the interaction between receptor-binding domain viral spike glycoprotein (RBD) and cell surface receptor angiotensin-converting enzyme-2 (ACE2). Percentage inhibition was calculated for each sample, and samples with a percentage inhibition ≥ 30% were considered to have neutralizing antibodies according to manufacturers' instructions and similar studies [8,9]. The seroprevalence of IgG antibodies and neutralizing antibodies were analyzed in the study population. IgG antibody levels were analyzed based on the S/Co ratio, while neutralizing antibody levels were analyzed using the percentage inhibition observed. The seroprevalence and antibody levels of IgG and neutralizing antibodies among different subcategories were compared. The Pearson chi-square test was used to compare the seroprevalence among subcategories, while the one-way ANOVA test was used to compare antibody levels.

The S/Co ratio of IgG antibodies for selecting donors for convalescent plasma donation differed in different guidelines. As per manufacturer instruction, reactivity is S/Co of 1.00; as per the Ministry of Health & Family Welfare, Government of India guideline, dated 22 April 2021, the cut-off was ≥ 3.5 [10]; and as per the AABB toolkit for high titer donors, the cut-off was ≥ 9.5 [11]. Based on these values, four categories were made. The first category was S/Co (< 1.00), the second was S/Co (1-3.49), the third was S/Co (≥ 3.5-9.49), and the fourth was S/Co (≥ 9.5). A comparison of neutralization antibody levels was made between these categories. The demographic data were collected in the form of whether they were accepted for donation or deferred, age, gender, occupation, type of donation, residence, previous history of COVID-19 infection, COVID-19 vaccination status, kind of vaccination, history of contact with a known COVID-19 patient, and blood group.

## Results

In the blood donor population, the seroprevalence of anti-SARS-CoV-2 IgG antibodies was 82.75% (576 reactive out of 696 tested), while neutralizing antibodies were 89.59% (241 reactive out of 269 tested). The detailed seroprevalence, along with IgG and neutralizing antibody levels in different subcategories of the study population with statistical comparison, is shown in Table 1.

**Table 1:** Seroprevalence of anti-SARS-CoV-2 IgG antibodies and neutralizing antibodies with respect to donor demographics *HCW - Healthcare Worker, **FLW - Frontline Worker Data have been represented as numbers (N), percentage (%), mean ±SD, and p value, which is considered significant at p <0.05.

Variable	Subcategory of the variable	Donors tested for SARS CoV-2 IgG (N)	SARS CoV-2 IgG reactive donors (N)	Percentage prevalence (%)	Mean S/CO ± SD confidence interval) (lower, upper)	p value of the difference in reactivity (p value of the difference in mean S/CO)	Donors tested for neutralizing antibodies (N)	Neutralizing antibodies reactive donors (N)	Percentage prevalence (%)	Mean percentage inhibition ± SD confidence interval) (lower, upper)	p value of the difference in reactivity (p value of the difference in mean percentage inhibition)
		696	576	82.75	8.59 ± 6.41 (8.12, 9.07)		269	241	89.59	74.82 ± 32.12 (70.97, 78.67)	
Donation status	Accepted	677	563	83.16	8.64 ± 6.42 (8.15, 9.12)	0.093 (0.302)	267	240	89.88	75.10 ± 31.85 (71.27, 78.92)	0.066 (0.089)
	Deferred	19	13	68.42	7.10 ± 6.42 (4.19, 10.00)		02	01	50	73.53 ± 29.55 (72.25, 74.82)	
Age in years	18-30	363	304	82.38	8.56 ± 6.39 (7.90, 9.22)	0.337 (0.099)	143	126	88.11	72.44 ± 33.69 (66.87, 78.01)	0.476 (0.434)
	31-45	276	222	80.43	8.29 ± 6.35 (7.54, 9.05)		95	88	92.63	77.69 ± 30.18 (71.57, 83.80)	
	46-65	56	49	87.5	10.31 ± 6.74 (8.51, 12.12)		31	27	87.09	76.90 ± 30.60 (65.67, 88.12)	
Gender	Male	678	559	82.44	8.47 ± 6.36 (7.99, 8.95)	(0.184) 0.02	253	227	89.72	74.85 ± 32.41 (70.85, 78.86)	0.778 (0.950)
	Female	18	17	94.44	13.15 ± 6.93 (9.70, 16.60)		16	14	87.50	74.33 ± 27.97 (59.42, 89.23)	
Occupation	HCW*	61	52	85.24	10.44 ± 6.94 (8.67, 12.22)	<0.001 (<0.001)	33	30	90.90	60.19 ± 40.21 (45.93, 74.45)	<0.001 (<0.001)
	FLW**	47	45	95.74	13.02 ± 5.76 (11.32, 14.7)		22	21	95.45	90.48 ± 17.72 (82.62, 98.33)	
	Business	113	98	86.72	9.27 ± 6.53 (8.05, 10.48)		46	42	91.30	76.49 ± 29.92 (67.61, 85.38)	
	Labor	116	91	78.44	6.59 ± 5.43 (5.59, 7.59)		35	32	91.42	69.65 ± 34.17 (58.09, 81.22)	
	Job work	246	213	86.58	9.18 ± 6.02 (8.42, 9.93)		96	88	91.66	82.18 ± 26.79 (76.75, 86.71)	
	Housework	48	20	41.66	1.57 ± 2.94 (0.72, 2.43)		13	5	38.46	28.80 ± 26.92 (12.52, 45.07)	
	Student	64	56	87.50	9.15 ± 6.35 (7.57, 10.74)		24	23	95.83	80.62 ± 24.65 (70.21, 91.03)	
Type of donation	Voluntary	131	112	85.49	9.39 ± 6.35 (8.29, 10.49)	0.353 (0.117)	48	41	85.41	62.97 ± 40.36 (51.25, 74.70)	0.296 (0.005)
	Replace ment	564	463	82.09	8.41 ± 6.42 (7.88, 8.95)		221	200	90.49	77.38 ± 29.53 (73.47, 81.29)	
Residence	Urban	497	414	83.29	8.70 ± 6.49 (8.13, 9.28)	0.620 (0.497)	212	189	89.15	73.73 ± 32.95 (69.28, 78.18)	0.648 (0.283)
	Rural	197	161	81.72	8.34 ± 6.22 (7.46, 9.21)		57	52	91.22	78.89 ± 28.69 (71.27, 86.50)	
Previous infection of COVID-19	Absent	661	546	82.60	8.53 ± 6.40 (8.04, 9.02)	(0.635) 0.225	241	214	88.79	76.38 ± 30.60 (72.51, 80.26)	0.211 (0.001)
	Present	35	30	85.71	9.88 ± 6.60 (7.61, 12.15)		28	27	96.42	61.32 ± 41.32 (45.30, 77.34)	
Vaccination history	Not vaccinated	342	256	74.85	6.16 ± 5.74 (5.55, 6.77)	<0.001 (<0.001)	111	97	87.38	67.81 ± 34.58 (61.34, 74.29)	0.321 (0.002)
	Vaccinated	354	320	90.3	10.95 ± 6.15 (10.30, 11.95)		158	144	91.13	79.79 ± 29.36 (75.17, 84.40)	
Type of vaccination (these data are only for vaccinated donors)	Covishield vaccinated	299	276	92.30	11.53 ± 5.93 (10.85, 12.21)	0.004 (<0.001)	129	119	92.24	81.79 ± 28.60 (76.81, 86.78)	0.301 (0.070)
	Covaxin vaccinated	55	44	80.00	7.77 ± 6.40 (6.04, 9.50)		29	25	86.20	70.87 ± 31.54 (58.87, 82.86)	
Contact with known COVID-19 Patient	No	669	552	82.51	8.45 ± 6.36 (7.96, 8.93)	0.039 (0.002)	252	225	89.28	74.15 ± 32.57 (70.12, 78.18)	0.528 (0.452)
	Yes	27	24	88.88	12.27 ± 6.70 (9.63, 14.92)		17	16	94.11	84.77 ± 22.91 (72.99, 96.55)	
Blood group	A	180	154	85.55	8.75 ± 6.39 (7.80, 9.69)	0.105 (0.559)	72	66	91.66	73.43 ± 32.66 (65.75, 81.11)	0.815 (0.132)
	B	233	198	84.97	8.97 ± 6.10 (8.18, 9.76)		94	85	90.42	80.85 ± 27.73 (75.17, 86.53)	
	AB	65	55	84.61	8.33 ± 6.55 (6.71, 9.96)		16	14	87.50	72.70 ± 37.97 (48.17, 87.23)	
	O	218	169	77.52	8.15 ± 6.71 (7.25, 9.04)		87	70	80.45	65.85 ± 34.33 (63.53, 78.16)	

The statistically significant difference in seroprevalence of IgG antibodies was present in categories of occupation (frontline workers), vaccination (vaccinated), and type of vaccination category (Covishield). In contrast, the statistically significant difference in IgG levels was found in the categories of gender (female), occupation (frontline workers), vaccination (vaccinated), type of vaccination (Covishield), and contact with known COVID-19 patients (contact present). The statistically significant difference in seroprevalence of neutralizing antibodies was present only in the occupation category (frontline worker). In contrast, the statistically significant difference in neutralizing antibody levels was found in the categories of occupation (frontline worker), type of donation (replacement donation), and vaccination (vaccinated) categories.

Donors vaccinated for COVID-19 showed a higher seroprevalence of 90.3% (320 out of 354), while donors with previous infection of COVID-19 showed a seroprevalence of 85.7% (30 out of 35). At the same time, the development of neutralization antibodies was higher in the donors who had a previous infection of SARS-CoV-2 (96.4%; 27 out of 28) than in vaccinated donors (91.1%, 144 out of 158). The median duration of infection date and day of testing in the donors with previous SARS-CoV-2 infection (N=35) was 195 days. On the other hand, the median duration of the vaccinated donors (N=354) on the day of vaccination and the day of testing was 112 days. Vaccination was more positively correlated with generating immune antibodies (Pearson correlation coefficient=0.206) than previous COVID-19 infections (Pearson correlation coefficient=0.018). In contrast, the previous COVID-19 infection was more positively correlated with the generation of neutralization antibodies (Pearson correlation coefficient=0.076) compared to vaccination (Pearson correlation coefficient=0.060).

**Figure 1:**
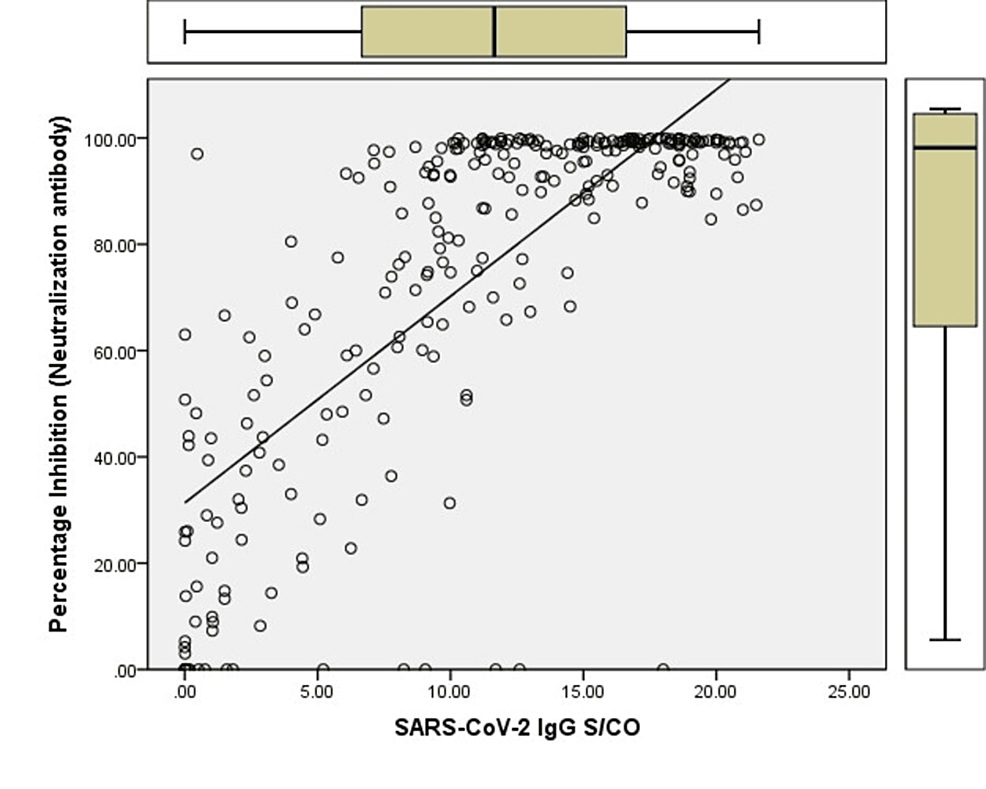
Correlation between SARS-CoV-2 IgG S/CO and percentage inhibition (neutralization antibodies) The X-axis shows the signal/cut-off of the SARS-CoV-2 IgG antibody, and the Y-axis shows the percentage inhibition of the neutralization antibody Pearson correlation coefficient=0.762

The correlation analysis between anti-SARS-CoV-2 IgG and neutralizing antibody levels showed a positive correlation (Pearson correlation coefficient=0.762) (Figure 1).

**Figure 2:**
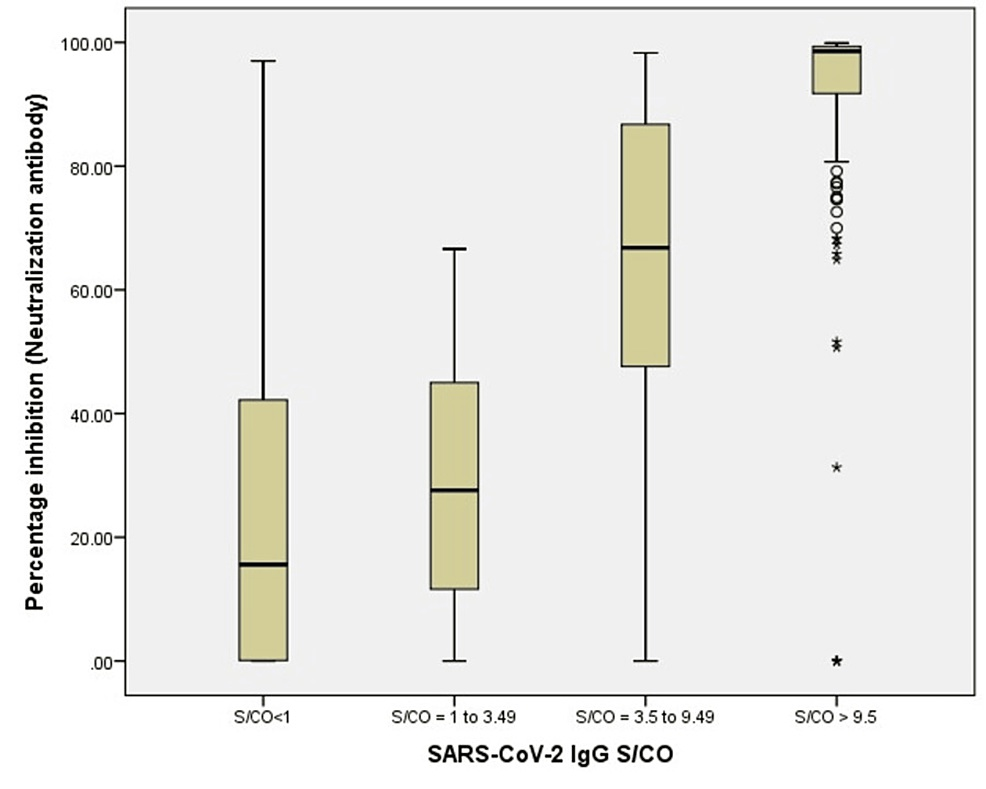
Bar diagram showing the percentage inhibition (neutralization antibodies) and SARS-CoV-2 IgG S/CO The X-axis shows four categories of the signal/cut-off of the SARS-CoV-2 IgG antibody, and the Y-axis shows the percentage inhibition of the neutralization antibody.

The neutralization antibody seroprevalence analysis was also done in the four categories based on the S/Co of IgG antibodies. In the first category of S/Co (< 1.00), 24 donors were tested for neutralization antibodies, and 10 (41.66%) had neutralization antibodies. In the second S/Co (1.00-3.49) category, 23 donors were tested for neutralization antibodies, and 13 (56.5%) had neutralization antibodies. In the third category of S/Co (3.50-9.50), 51 donors were tested, and 47 (92.15%) had neutralization antibodies. In the fourth category of S/Co (≥ 9.5), 171 donors were tested for neutralization antibodies, and almost all were reactive for neutralization antibodies (Figure 2).

## Discussion

The seroprevalence of IgG antibodies in the present study was 82.75%, corresponding to the national serosurvey data of that period on a similar population (73.1% of the Uttarakhand state of India) [12]. Published systemic review and meta-analysis of 53 studies with 905,379 participants from March 1, 2020, to August 11, 2021, on the Indian population showed pooled seroprevalence of 69.2% in the general population in the second wave in India [13]. The blood donor population is a subset of the people that excludes extreme age (less than 18 years of age and greater than 65 years of age) but is representative of the active mobile population who came forward for the generous act of blood donation. There is some unique advantage of assessing seroprevalence in the blood donor population for getting an idea of seroprevalence in the general population because, logistically, it is more straightforward, accessible, inexpensive, and convenient as compared to selecting, recruiting voluntary donors, taking different samples, taking consent with following COVID-19 precautions, etc. [14]. As per India's demography, the blood donors' age group represents nearly 70% of the total population and 90% of the adult population [15]. Other studies have shown the importance of estimating seroprevalence against the SARS-CoV-2 infection in the blood donor population for assessing seroprevalence in the general population by defining a particular model considering parameters such as infectivity, susceptibility of different age groups, recovery rate, preclinical and clinical infectiousness, etc. [16,17]. Seroprevalence in the general population may be higher than the blood donor population by the mathematical formulas. However, a study by Stone et al. showed higher seroprevalence in the blood donor population than in the general population [18]. Although the current study did not estimate seroprevalence in the general population, the seroprevalence in the blood donor population was compared with the seroprevalence in the general population by the systemic review by Jahan et al. of that time and found higher in blood donors [13].

A study by Chunchu et al. found statistically higher IgG antibody levels in 31-45 years age group blood donors. In contrast, no statistically significant difference was found in age in the present study [9]. One of the reasons may be that vaccination was started early in the senior population and moved to the adult population. Seroprevalence of IgG antibodies was higher in vaccinated than non-vaccinated donors, which agreed with other studies [19,20]. The higher immune response of IgG antibodies in Covishield-vaccinated donors versus Covaxin-vaccinated donors was similar to the ICMR studies, but a non-significant difference in neutralization antibodies seroprevalence in both types of vaccine is a new finding. Covishield targets spike protein, while Covaxin-inactivated virus could be the reason for the difference in IgG antibodies' seroprevalence as the method of IgG testing in the study targets spike-binding antibodies [21,22].

The higher seroprevalence of neutralization antibodies in the study population compared to spike-binding antibodies (IgG) may be because of the different sensitivity levels of different methods. As per the literature [23], IgM and IgG1 antibodies constitute most of the neutralization antibodies, which could also be the reason for the higher prevalence of neutralization antibodies. The study could not conclude the same because the specificity (whether IgM, IgG, or IgA) of the neutralization antibodies was not analyzed in the current research. Another fact is that, in the non-vaccinated individuals, assuming the source of immunization is asymptomatic natural SARS-CoV-2 infection, 74.65% have spike-binding antibodies, while 87.38% have neutralization antibodies. On the other hand, in the vaccinated category, the same are 90.3% and 91.13%, respectively. This showed that asymptomatic natural infection generates more neutralization antibodies than IgG antibodies, while, in the vaccination category, both are similar but higher than the natural infection. Previous SARS-CoV-2 infection more positively correlated with neutralization antibodies, while vaccination more positively correlated with the generation of IgG antibodies. This showed a difference in immune response in infection (either symptomatic or asymptomatic) and vaccination category. Another interesting finding is that, in Covishield-vaccinated individuals, seroprevalence of spike-binding and neutralization antibodies were the same (92.3%). In contrast, in Covaxin-vaccinated individuals, neutralization antibody prevalence was higher (86.2%) than spike-binding antibodies (80%). The above observations can conclude that asymptomatic natural infections and Covaxin-vaccinated individuals produced more neutralization antibodies than spike-binding antibodies, while Covishield-vaccinated produced both in similar proportions. The study by Das et al. mentioned that Covaxin is more effective against variants because of its ability to make multiple antibodies against various epitopes [23].

In the current study, 41.66% of the donors with spike-binding antibody negativity still have neutralization antibody positivity. The individuals' reactivity difference in the generation of spike-binding or neutralization antibodies may also explain this. Studies have also shown that various neutralization antibodies provide immunity by different mechanisms [24,25]. Although the current study did not analyze the biochemistry of the antibody characteristics, the finding of neutralization antibodies in IgG antibody-negative donors showed their protection against the SARS-CoV-2 infection. Another explanation may be due to the difference in sensitivity of the assays. Neutralizing antibodies protect against reinfection and provide herd immunity. High-titer-neutralizing antibodies are capable of neutralizing virus variants [26,27]. This high titer-neutralizing antibody-containing plasma can prepare hyperimmunoglobulin, monoclonal antibodies, and lyophilized products [28].

Doctors, nurses, community health workers, sanitation workers, police, volunteers, and ambulance drivers, among many others, valiantly stood behind the Government in the country's crisis and were honored by the Government of India as frontline workers (FLWs) [29]. Doctors, nurses, and all supporting hospital staff are termed healthcare workers (HCWs). In the present study, FLWs, excluding HCWs, showed the highest immune response in the occupation category. It may reflect that exposure is the primary source of immunization because all categories have an equal chance of vaccination. HCWs being trained also take more precautions for COVID-19 prevention. The lowest immune response was found in donors engaged in housework, making them more susceptible to infection. There is also a significant difference in the donors with IgG antibody levels who have documented exposure to known SARS-CoV-2-infected patients. In this study, 35 donors documented SARS-CoV-2 infection (17 were also vaccinated: Covishield (12) and Covaxin (five)), and 354 were vaccinated (209 for a single dose and 141 for both doses).

Interestingly, 328 blood donors (47.12% of the total study population) with no vaccination history nor documented SARS-CoV-2 infection had IgG antibodies against SARS-CoV-2 infection, showing that natural immunity protected India during the second wave. In the current study, the median duration of infection or vaccination to the day of testing was analyzed, and it was found to be less in vaccinated individuals. It was tough to say what had led to the development of immunity as in community spread; the individual is repeatedly exposed. Thus, even after vaccination or previous SARS-CoV-2 infection, donors may be exposed again later. Therefore, the impact of duration was not analyzed in detail.

Neutralizing antibody titers can be estimated using the plaque reduction neutralization test (PRNT), a gold standard test. The requirement of dedicated infrastructure and technical expertise is necessary for PRNT as it requires handling live viruses. The blocking ELISA detection tool, which mimics the virus neutralization process after validation, can be used to detect neutralization antibodies. Studies have shown that S/Co from various anti-SARS-CoV-2 antibody assays provide rough estimates of neutralizing antibody titers. A study done by Chunchu et al. found that donor samples having S/Co ≥ 9.5 have significantly higher neutralizing capacity (> 68%) as compared to S/Co ≤ 9.5 [9]. In the present study, almost all donors' S/Co ≥ 9.5 had neutralizing antibodies. In comparison, 92.15% of the donors' S/Co samples of S/Co ≥ 3.5 had neutralizing antibodies. The method of SARS-CoV-2 IgG antibodies by S/Co in both studies was the same (chemiluminescence-based assay of ortho clinical diagnostics) while neutralizing antibody assay by ELISA; although it is based on the same principle, it belongs to different manufacturers. The former study was done in the first wave of the pandemic, while the current study was done in the second wave. The present study also showed a positive correlation between S/Co and neutralizing antibodies (Pearson correlation coefficient=0.762). One practical problem in neutralizing antibodies is that these are tested in batches in block ELISA, which is time-consuming. Other simple methods can test IgG immune antibodies. Establishing a correlation between IgG and neutralizing antibodies will help predict neutralizing antibody levels by testing only immune antibodies. This may be helpful in a blood bank setting where high titer plasma may be isolated from the rest for consideration as CP by testing in the same automated equipment that tests viral transfusion-transmitted infection in the shortest turnaround time.

Studies showed that seroprevalence in the first wave was 20.7%, while, in the second wave, it was above 69.2% [13]. Assuming seroprevalence of the first wave as a baseline, testing blood donors at the start of the second wave might lead to readily available convalescent plasma of 20% of the donated blood. It will be hypothesized that, instead of dedicated searching for a convalescent plasma donor, if seroprevalence is high, then it is easy to get a convalescent plasma donor in the blood donor population. The donated plasma can be used as convalescent plasma, especially higher S/CO, which can be validated with neutralizing antibodies in that population without much logistical difficulty. This will avoid searching, selecting, and recruiting a convalescent plasma donor during the pandemic. This plasma may be used not only as a treatment modality but also to plan properly randomized control trials in any stage of disease, depending on the study; for example - studies have suggested the effect of convalescent plasma in the early stage of disease [2,30] logistically if convalescent plasma is not readily available with the blood center. There is usually a delay in providing convalescent plasma therapy in the early stage of the disease. The cost of testing will be an issue, but testing selective donor populations including exposed persons such as FLWs and those previously infected or exposed with known SARS-CoV-2 persons may be more cost-effective. These studies suggested using whole blood-derived convalescent plasma as an alternative to apheresis-derived. The present study recommends testing all whole blood donors at a particular time, for example, when seroprevalence reaches a higher level. This may be helpful in case of a new mutant of the SARS-CoV-2 or epidemic or pandemic by any new virus.

This current study did not evaluate the in vivo effectiveness of whole blood-derived CP. Another limitation was the lack of invitro tests to assess the neutralizing antibody potency of convalescent plasma against different variants.

## Conclusions

The current study found higher seroprevalence in the blood donor population than published seroprevalence in India's second pandemic wave. The seroprevalence of neutralizing antibodies was higher than the seroprevalence of the anti-SARS-CoV-2 IgG antibodies in the donors who had previous infection of COVID-19. On the other hand, vaccinated donors showed similar immune responses for neutralizing antibodies and anti-SARS-CoV-2 IgG antibodies. Similar neutralizing antibodies in Covaxin- and Covishield-vaccinated donors showed identical efficacy in generating neutralizing antibodies, although immune IgG antibodies were found more in the Covishield-vaccinated group. Frontline workers showed maximum immunity, while persons working from the house showed the most minor, making them more susceptible to infection. In the current study, 328 donors (47.12%) of the 696 screened donors were neither vaccinated nor had previous SARS-CoV-2 infection, but many had antibodies. There was a good correlation between anti-SARS-CoV-2 IgG antibodies and neutralizing antibodies. One-fourth of the study population showed high titer antibodies, and almost all donors of S/Co ≥ 9.5 had neutralizing antibodies. Convalescent plasma therapy may be experimented with in case of future epidemics or pandemics by mutant SARS-CoV-2 or any new virus by screening donated blood in the blood center in case of unavailable established treatment options when seroprevalence in the general population reaches a higher level by screening highly exposed donors having high titer antibodies.
